# Causal association between inflammatory bowel disease and IgA nephropathy: A bidirectional two-sample Mendelian randomization study

**DOI:** 10.3389/fgene.2022.1002928

**Published:** 2022-11-16

**Authors:** Mofan Xiao, Yan Ran, Jiayuan Shao, Zhangni Lei, Yuling Chen, Yingchao Li

**Affiliations:** ^1^ Department of Gastroenterology, The First Affiliated Hospital of Xi’an Jiaotong University, Xi’an, China; ^2^ Department of Gastroenterology, The Second Affiliated Hospital of Xi’an Jiaotong University, Xi’an, China

**Keywords:** inflammatory bowel disease, ulcerative colitis, Crohn’s disease, IgA nephropathy, Mendelian randomization, causality

## Abstract

**Background:** An association between inflammatory bowel disease (IBD) [which includes ulcerative colitis (UC) and Crohn’s disease (CD)] and IgA nephropathy (IgAN) has been discovered in observational studies, but the causal relationship is still unknown. The aim of this study was to clarify the causal link between IBD (which includes UC and CD) and IgAN *via* a two-sample Mendelian randomization (MR) analysis.

**Methods:** Eligible single-nucleotide polymorphisms (SNPs) were selected as instrumental variables (IVs) for analyses and were obtained from the publicly available genome-wide association study (GWAS) summary statistics. Inverse-variance weighting (IVW), Mendelian randomization–Egger (MR-Egger) regression, the Mendelian randomization pleiotropy residual sum and outlier (MR-PRESSO) test, and the weighted median were utilized to obtain the results. The MR-PRESSO test and MR-Egger regression were also performed to detect and correct horizontal pleiotropy. The Cochran’s Q test and “leave-one-out” analysis were also conducted to assess the stability and reliability of the MR results.

**Results:** This study found that IBD, UC, and CD all had significant positive causal effects on IgAN risk (IBD: OR = 1.58, 95% CI 1.15–2.16, *p* = 4.53 × 10^–3^; UC: OR = 1.55, 95% CI 1.14–2.11, *p* = 4.88 × 10^–3^; CD: OR = 1.57, 95% CI 1.21–2.03, *p* = 5.97 × 10^–4^). No significant horizontal pleiotropic effect was found for the causal association between IBD, UC, CD, and the risk of IgAN. Cochran’s Q test identified no evidence of heterogeneity for the IV estimates. The “leave-one-out” sensitivity analysis also revealed that the MR results were robust.

**Conclusion:** The results of this two-sample MR analysis supported that IBD, UC, and CD were causally associated with the risk of IgAN, while there was no sufficient evidence for the causal effect of IgAN on IBD, UC, or CD. Our findings provide theoretical support and a new perspective for the diagnosis and treatment of these two diseases.

## 1 Introduction

Immunoglobulin A nephropathy (IgAN) is the most common primary glomerulonephritis worldwide and can progress to chronic kidney disease and renal failure (Pattrapornpisut et al., 2021). In the early stage, it is characterized by asymptomatic hematuria with minimal proteinuria, which can be detected by screening programs. As the disease progresses, a proportion will develop hypertension, significant proteinuria, progressive glomerulonephritis, and even end-stage kidney disease (ESKD). Another phenotype presents recurrent macroscopic hematuria and is associated with a favorable prognosis in the short term, commonly in patients aged under 40 years. The incidence of IgAN varies from 0.54 to 10.5 per 100,000 population per year, with a high prevalence in young people aged between 20 and 40 years (Schena and Nistor, 2018). IgAN is considered an immune-mediated disease influenced by a combination of genetic and environmental factors. The typical pathological feature of IgAN is the deposition of polymeric and galactose-deficient IgA1 (Gd-IgA1) in the glomerular mesangium. Kiryluk et al. (2011) indicated that circulating levels of Gd-IgA1 were a heritable trait and were increased in patients with IgAN and their first-degree relatives. Genome-wide association study (GWAS) data sets also identified multiple susceptibility loci for IgAN and several risk alleles associated with intestinal epithelial barrier maintenance and mucosal immunity (Yu et al., 2011; Kiryluk et al., 2014; Li et al., 2020).

Inflammatory bowel disease (IBD), which includes ulcerative colitis (UC) and Crohn’s disease (CD), is a chronic and idiopathic inflammatory disease of the gastrointestinal tract (Torres et al., 2017; Ungaro et al., 2017). Mucosal inflammation in UC mainly affects the rectum to the proximal segments of the colon, whereas in CD, all segments of the gastrointestinal tract can be affected, with the terminal ileum being the most common site. The common features of IBD are abdominal pain, chronic diarrhea, weight loss, and varying degrees of systemic symptoms (Flynn and Eisenstein, 2019). Some patients with IBD might exhibit extraintestinal manifestations, such as arthritis, spondyloarthropathy, episcleritis, uveitis, erythema nodosum, and primary sclerosing cholangitis. With societies in newly industrialized countries becoming more westernized, the incidence of IBD is increasing worldwide. It has been reported that the incidence of UC ranges from 0.15 to 57.9 per 100,000 person-years, while the incidence of CD ranges from 0.09 to 23.82 per 100,000 person-years (Ng et al., 2017). As a multifactorial disease, the pathogenic etiology of IBD is still incompletely understood. At present, it is generally believed that chronic intestinal inflammation is caused by an interaction of genetic susceptibility, excessive immune response, gut microbiota, and various environmental triggers. As shown by existing evidence, the first-degree relatives of patients with IBD have a five-fold increased risk of developing IBD (Flynn and Eisenstein, 2019). Hundreds of genetic loci have also been shown to be associated with IBD *via* GWAS (Liu et al., 2015). Moreover, a nationwide cohort study in Sweden has revealed an association between IgAN and IBD (Rehnberg et al., 2021).

Mendelian randomization (MR) can be used to explore the causal effect of exposure on outcomes by taking the differences in genotype as instrumental variables (IVs) (Emdin et al., 2017). Due to the natural random assortment of genetic variation during meiosis, Mendelian randomization has been proposed as a method analogous to classic randomized controlled trials (Emdin et al., 2017; Wu et al., 2020). Because genotypes appear before diseases and are largely independent of the postnatal lifestyle and environmental factors, MR is free from confounding factors and can avoid the biasing effect of reverse causality. As the large-scale GWAS reliably identifies genetic variants, MR has been successfully adopted for investigating causal links.

In this two-sample MR study, significant and independent single-nucleotide polymorphisms (SNPs) were chosen as IVs to clarify the causal association at the genetic level using the GWAS data. The aims of this study are to clarify if IBD, UC, and CD have the potential causal effect on IgAN and whether the reverse causal effect exists.

## 2 Material and methods

### 2.1 Data source

The genetic data of IBD, UC, CD, and IgAN originated from the large published GWAS. All participants were of European ancestry. The summary statistics of exposures included IBD (*N* = 31,665 cases and 33,977 controls), UC (*N* = 6,968 cases and 20,464 controls), and CD (*N* = 5,956 cases and 14,927 controls). Liu et al. (2015) elucidated the details of the data used for IBD, UC, and CD. For IgAN, the GWAS summary statistics included 977 subjects with IgAN and 4,980 disease-free controls (Feehally et al., 2010).

### 2.2 Selection of instrumental variables

In MR, there are three key assumptions that are required to be fulfilled: first, the SNPs must be associated with the exposure; second, the SNPs should be randomly distributed and independent of any confounders; and third, the SNPs affect the outcome only through the exposure (Sekula et al., 2016). When SNPs meet these three stringent assumptions, they are treated as IVs. In this study, we extracted SNPs that were significantly associated with exposures with genome-wide significance (*p* < 5 × 10^–8^) as IVs. Then, to ensure that all the instrumental SNPs had independent inheritance, we used a clumping algorithm with a cut-off of *r* ^2^ = 0.1. The SNPs in linkage disequilibrium (*r* ^2^ > 0.1) were excluded. Furthermore, the Mendelian randomization pleiotropy residual sum and outlier (MR-PRESSO) test was used to identify and remove pleiotropic variants that might cause significant pleiotropy. The F-statistic was used to measure the strength of each instrument, and SNPs with an F value < 10 labeled “weak instruments” were excluded (Burgess et al., 2017). Additionally, *R* ^2^ was calculated to explain the proportion of the variation of IBD, UC, and CD by each IV (Burgess and Thompson, 2011). Finally, a calculation of statistical power was performed using mRnd (https://sb452.shinyapps.io/power/), developed by Brion et al. (2013).

### 2.3 Effect size estimate

The causal associations between exposures (IBD, UC, and CD) and outcomes (IgAN) were mainly estimated with inverse-variance weighting (IVW), Mendelian randomization–Egger (MR-Egger) regression, and the weighted median. The MR-PRESSO test was also used to estimate the causal relationships between IBD, UC, and CD and IgAN to acquire robust causal effects. The IVW method that combines the Wald ratios of each SNP provides the most precise estimates of the causal effect when each genetic variation satisfies the assumption of the IVs (Burgess et al., 2013). MR-Egger regression is used to perform a weighted linear regression of the outcome coefficients on the exposure coefficients. The intercept of MR-Egger regression can estimate the presence of directional pleiotropy. The statistical significance of MR-Egger regression might be inaccurate and strongly influenced by unrelated genetic variations (Bowden et al., 2016b). The weighted median provides valid MR estimates, with more than 50% weights coming from effective IVs in the analysis. Compared with the MR-Egger regression, the weighted median has distinct superiorities for its improved power of causal effect detection (Bowden et al., 2016a). However, when the percentage of horizontal pleiotropic variants is high (≥50%), the opposite conclusion is reached. Hence, MR-Egger regression provides a more robust estimate of potential violations of the MR assumptions (Verbanck et al., 2018).

### 2.4 Sensitivity analyses

To meet MR assumptions, we conducted multiple sensitivity analyses to assess heterogeneity and pleiotropy within the genetic instruments. Pleiotropy refers to a locus affecting multiple phenotypes, and a genetic variant is associated with more than one phenotype, which is a violation of MR assumption 3. MR-PRESSO and MR-Egger regression were performed to assess the potential pleiotropic effect of the SNPs used as IVs after removing the outliers. The “leave-one-out” sensitivity analysis is an algorithm to ensure the reliability of the association of the SNPs with exposure. The algorithm could reanalyze the results and draw a forest map by leaving out each SNP in turn.

For the heterogeneity analysis, Cochran’s Q test and I^2^ statistics were performed. The Cochran Q statistic was calculated as the weighted sum of the squared differences between each SNP effect and the summed effect across all SNPs. The I^2^ statistic values indicated the expected relative bias of the MR-Egger causal estimate (Bowden et al., 2016b).

The results are presented as odds ratios (ORs) and 95% confidence intervals (CIs). A *p*-value less than 0.017 (0.05/3 adjusted with the Bonferroni method) was considered statistically significant. All statistical analyses were conducted using the R (version 4.1.2) software with the “Two-Sample MR” package. This analysis used the published study data or publicly available GWAS data and therefore did not require an ethics committee approval. In all original studies, ethical approvals were obtained.

## 3 Results

### 3.1 Selected single-nucleotide polymorphisms of the study

A total of 56, 33, and 55 significant (*p* < 5 × 10^–8^) and independent (*r* ^2^ < 0.1) SNPs were selected as IVs for IBD, UC, and CD, respectively (Table 1). The F-statistics for every instrument exposure association was much greater than 10 in this study, with average F values of 53.04, 64.64, and 62.58 for IBD, UC, and CD, respectively. The F-statistics ranged from 30 to 213 for IBD, 29 to 342 for UC, and 30 to 348 for CD, which indicated that the strength of the variables satisfied the relevance assumption of MR. Furthermore, the instrument bias was weak, which could not substantially influence the estimations of the causal effect. Detailed information about the genetic variants is listed in Supplementary Tables S1–S3.

**TABLE 1:** Causal effects of IBD, UC, and CD on IgA nephropathy based on MR.

Exposure	MR method	IgA nephropathy				
		SNPs	OR	95% CI	*p*-value	Statistical power (%)
IBD	IVW	56	1.58	1.15–2.16	4.53 × 10^–3^	100
IBD	MR-Egger	56	1.33	0.43–4.13	0.623	
IBD	Weighted median	56	1.52	0.98–2.35	0.063	
UC	IVW	33	1.55	1.14–2.11	4.88 × 10^–3^	99
UC	MR-Egger	33	1.37	0.54–3.48	0.507	
UC	Weighted median	33	1.54	1.02–2.33	0.039	
CD	IVW	55	1.57	1.21–2.03	5.97 × 10^–4^	100
CD	MR-Egger	55	0.90	0.42–1.96	0.794	
CD	Weighted median	55	1.42	1.03–1.97	0.034	

IBD, inflammatory bowel disease; UC, ulcerative colitis; CD, Crohn’s disease; MR, Mendelian randomization; SNP, single-nucleotide polymorphism; OR, odds ratio; CI, confidence interval; IVW, inverse-variance weighting; MR-Egger, Mendelian randomization–Egger.

### 3.2 Causal effect of inflammatory bowel disease on immunoglobulin A nephropathy

**FIGURE 1:**
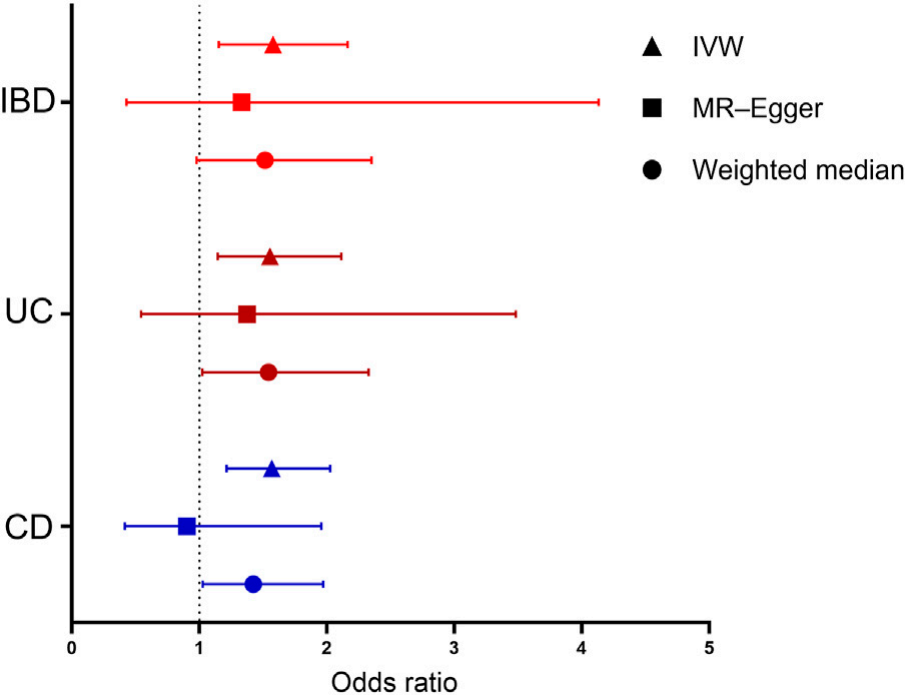
Forest plot for the causal effects of inflammatory bowel disease (IBD), ulcerative colitis (UC), and Crohn’s disease (CD) on IgA nephropathy (IgAN). Each point represents the combined causal estimate using all SNPs together in a single instrument with the IVW (triangle), MR-Egger (square), and weighted median (circle) methods. Horizontal lines represent the 95% confidence interval (red: IBD, brown: UC, and blue: CD). (IBD, inflammatory bowel disease; UC, ulcerative colitis; CD, Crohn’s disease; SNP, single-nucleotide polymorphism; IVW, inverse-variance weighting; MR-Egger, Mendelian randomization–Egger.)

The MR analysis assessing the causal effect of IBD on IgAN is shown in Tables 1, 2, Figure 1, and Supplementary Figures S1–S4. The IVW method indicated that IBD had a positive causal effect on IgAN (OR = 1.58, 95% CI 1.15–2.16, *p* = 4.53 × 10^–3^) with 100% statistical power (Table 1; Figure 1). The MR-PRESSO analysis also supported the positive causal effect of IBD on IgAN (*p* = 0.006). Scatter plots, forest plots, and funnel plots of SNPs associated with IBD and IgAN are shown in Supplementary Figures S1–S3. Pleiotropy, heterogeneity, and sensitivity analyses were conducted for quality control. For pleiotropy, MR-Egger regression (intercept = 0.014, *p* = 0.760) and the MR-PRESSO global test (*p* = 0.159) revealed that horizontal pleiotropy was unlikely to bias the causality of IBD (Table 2). The Cochran Q-value and the I^2^-value indicated that there was no heterogeneity between the IV estimates (MR-Egger Q = 65.052, *p* = 0.144; IVW Q = 65.166, *p* = 0.164; I^2^ = 15.60%) (Table 2). The “leave-one-out” sensitivity analysis evaluated the influence of outlying or pleiotropic SNPs by leaving out each SNP in turn. The results revealed that the causal link between IBD and IgAN was not significantly affected by any one SNP (Supplementary Figure S4).

**TABLE 2:** Heterogeneity and pleiotropy analyses of IBD, UC, and CD with IgA nephropathy.

Exposure	MR-Egger				IVW		I^2^
	Intercept	Pleiotropy *p*-value	Cochran’s Q statistic	Heterogeneity *p*-value	Cochran’s Q statistic	Heterogeneity *p*-value	
IBD	0.014	0.760	65.052	0.144	65.166	0.164	15.60%
UC	0.015	0.785	41.618	0.096	41.719	0.117	23.30%
CD	0.065	0.144	70.103	0.058	73.008	0.043	26.04%

IBD, inflammatory bowel disease; UC, ulcerative colitis; CD, Crohn’s disease; MR, Mendelian randomization; MR-Egger, Mendelian randomization–Egger; IVW, inverse-variance weighting.

### 3.3 Causal effect of ulcerative colitis on immunoglobulin A nephropathy

The MR results regarding the causal relationship between UC and IgAN with great statistical power are shown in Tables 1-2, Figure 1, and Supplementary Figures S4–S6. After removing the SNPs (rs434841) that might cause pleiotropy with the help of the outlier test in MR-PRESSO analyses, no horizontal pleiotropy was identified according to the MR-Egger regression (intercept = 0.015, *p* = 0.785) (Table 2). Then, the IVW method results suggested that the per unit increase in the log-odds of having UC increase the risk of having IgAN was 0.55-fold (OR = 1.55, 95% CI 1.14–2.11, *p* = 4.88 × 10^–3^) (Table 1; Figure 1). The outlier-corrected MR-PRESSO analysis (*p* = 0.008) also found an analogous result (Table 1; Figure 1). Scatter plots, forest plots, and funnel plots of SNPs related to the causal effect of UC on IgAN are presented in Supplementary Figures S5–S7. The Cochran Q-value and the I^2^-value indicated that there was no heterogeneity between the IV estimates (MR-Egger Q = 41.618, *p* = 0.096; IVW Q = 41.719, *p* = 0.117; I^2^ = 23.30%) (Table 2). Moreover, the causal effect of UC on IgAN did not significantly fluctuate with any single SNP in the “leave-one-out” sensitivity analysis (Supplementary Figure S8).

### 3.4 Causal effect of Crohn’s disease on immunoglobulin A nephropathy

As shown in Table 1 and Figure 1, the IVW results found that CD was positively associated with IgAN (IVW: OR = 1.57, 95% CI 1.21–2.03, *p* = 5.97 × 10^–4^) with 100% statistical power. The positive causal relationship of CD with IgAN was also supported by the causal estimate in MR-PRESSO analysis (*p* = 0.001). Supplementary Figures S9–S11 show the scatter plots, forest plots, and funnel plots of SNPs for the causal effect of CD on IgAN. The MR-PRESSO analysis (*p* = 0.057) and MR-Egger regression analysis (intercept = 0.065, *p* = 0.144) suggested no evidence of horizontal pleiotropy. In addition, there was no heterogeneity (MR-Egger Q = 70.103, *p* = 0.058; IVW Q = 73.008, *p* = 0.043; I^2^ = 26.04%) across the IV estimates in this part (Table 2). Moreover, the results were also confirmed by the “leave-one-out” sensitivity test, suggesting that they were stable and reliable (Supplementary Figure S12).

### 3.5 Causal effects of immunoglobulin A nephropathy on inflammatory bowel disease, ulcerative colitis, and Crohn’s disease

Furthermore, this study also conducted MR analyses of the causal effects of IgAN on IBD, UC, and CD. Only one SNP (rs3115573) was selected to access the causal effects of IgAN on IBD, UC, and CD, respectively. According to the results of the Wald ratio method, IgAN was positively associated with IBD (OR = 1.06, 95% CI = 1.02–1.11, *p* = 0.008), UC (OR = 1.08, 95% CI = 1.02–1.14, *p* = 0.008), and CD (OR = 1.07, 95% CI = 1.01–1.12, *p* = 0.013). However, there were not enough SNPs available for heterogeneity or pleiotropy analyses, leading to unreliable results. Hence, these causal effects of IgAN on IBD, UC, and CD should be treated cautiously, and more studies are required to determine their causal relationships.

## 4 Discussion

To the best of our knowledge, this study has been considered the first to illustrate the causal effect of IBD (which includes UC and CD) on IgAN using MR analysis and summary statistics from GWAS. As the data from this study revealed, IBD, UC, and CD causally increased the risk of IgAN in individuals of European descent. However, there was no sufficient evidence supporting the inverse causal relationship between these two diseases.

IgAN is the most common type of primary glomerulonephritis worldwide, with an increasing incidence. An analysis of cohorts from Europe and North America found that the 5- and 10-year risks of a 50% decrease in eGFR or ESKD in IgAN patients were 11.2% and 26.8%, respectively (Barbour et al., 2016). The pathological mechanism of IgAN is complex and is yet to be fully elucidated. The “multi hit hypothesis” has been widely accepted to explain IgAN pathogenesis. In this hypothesis, aberrant Gd-IgA1 increased and was targeted by anti-glycan IgG autoantibodies, causing the formation of immune complexes. These immune complexes cannot be adequately cleared from circulation and deposit in the mesangium, which might activate the complement system and trigger glomerular inflammation (Pattrapornpisut et al., 2021).

Previous case reports and clinicopathologic series have shown the relationship between IBD and IgAN and discussed the pathophysiologic links. In a case study from Korea, it was reported that a patient with rapidly progressive IgAN, concurrently presented with CD exacerbation, demonstrating a direct link between the aggravation of renal function and intestinal symptoms. Moreover, both renal and inflamed colonic tissues were positive for IL-17 and responded to immunosuppressive therapy. This might indicate a potential common pathogenesis of these two diseases (Choi et al., 2012). Ambruzs et al. (2014) concluded that the frequency of IgAN in IBD was significantly higher than that in other native kidney biopsy specimens during the same period [20 of 83 (24%) *versus* 2,734 of 33,630 (8%)]. A cross-sectional study from China evaluated 33 renal biopsy specimens from patients with IBD, showing that more than half (66%) of the pathological lesions were IgAN (Zhao et al., 2021). Elaziz and Fayed (2018) included 896 patients with IBD in a study and found that 218 (24.3%) of them had developed renal manifestations, of whom 35 patients were diagnosed with IgAN. Furthermore, Rehnberg et al. (2021) launched a nationwide cohort study and found that IgAN patients had an increased risk of IBD both before and after a confirmed IgAN diagnosis. It has been reported that patients with IBD show increased serum IgA levels and an increased incidence of abnormal hematuria, while patients with IgAN present significantly higher intestinal permeability, which is related to increased hematuria, proteinuria, and serum levels of IgA (Wang et al., 2004; Papista et al., 2011).

Although the exact mechanisms linking IBD and IgAN are not fully understood, the gut–kidney axis hypothesis might potentially support the pathophysiological relationship. It was assumed that dysregulation of the interplay among intestinal immunity, microbiota, and diet could lead to the production of Gd-IgA1, which plays an important role in IgAN (Joher et al., 2022). Together with mucus and antimicrobial peptides, IgA forms the first line of defense against environmental and microbial antigenic exposures occurring in the intestinal mucosa (He et al., 2020). A disruption of the epithelial barrier is more likely to lead to exposure to immunogenic alimentary antigens and increased IgA production. Kovács et al. (1996) found that significantly higher intestinal epithelial permeability was associated with increased serum levels of IgA against food antigens, such as soy protein. Chronic intestinal inflammation contributes to persistent and excessive activation of toll-like receptors, which might eventually result in the overproduction of IgA1/Gd-IgA1 (He et al., 2020). Then, in patients with IBD, the overstimulation of mucosal B cells significantly shifts immunoglobulin production from IgA2 to IgA1 (Brandtzaeg et al., 2006). Moreover, the number of N-acetylgalactosamines in the O-linked oligosaccharides of IgA significantly decreased in CD patients and strongly correlated with clinical activity (Inoue et al., 2012). Additionally, Wang et al. (2004) demonstrated that abnormally activated T cells initiated severe intestinal inflammation that could result in the dysregulation of serum IgA levels, indicating that T-cell-mediated intestinal mucosal immunity was critical in the pathogenesis of IgAN. Hence, a complex interplay of mucosal inflammation, chronic immune stimulation, microbiota, abnormal IgA glycosylation, and dysregulated IgA production and transport might potentially influence the pathogenesis of IBD and IgAN. Furthermore, genetic studies have found that GWAS of patients with IgAN identifies risk loci involved in intestinal mucosal integrity and the immune network (Li et al., 2020).

In addition, patients with IgAN comorbid with IBD have accelerated disease progression, while the remission of bowel disease could also improve renal function. Rehnberg et al. (2021) confirmed that patients with IgAN and IBD had an increased risk of progression to ESKD when compared with patients with IgAN without IBD. The identification of IBD might be useful for predicting the risk of ESKD in patients with IgAN. In 1984, a French physician first reported IgAN associated with IBD (one patient with UC and one patient with CD). With symptomatic treatment of the intestinal disease, not only did hematuria subside but also did mesangial hyperplasia and IgA deposition disappear (Hubert et al., 1984). Since then, a growing number of studies have suggested that intestinal disease therapy is associated with improvements in renal manifestations (Onime et al., 2006; Filiopoulos et al., 2010). Smerud et al. (2011) suggested that enteric budesonide targeted to the ileocecal region could represent a new treatment of IgAN for reducing urine albumin excretion.

There are several limitations in this present research. First, a bidirectional two-sample MR analysis to identify the causal relationships between IgAN and IBD was attempted, but sufficient numbers of SNPs were not available to determine the causal effect of IgAN on IBD, UC, and CD, which led to biased results. Second, this analysis was based on GWAS data sets, making it difficult to stratify the causal effect by race, age, sex, or other risk factors. Although the evaluation of other IgAN cohorts would be ideal to confirm our findings, no other IgAN cohorts could be found in the GWAS data sets. As a result, we performed direct experiments on the causal relationships between IgAN and IBD, UC, and CD in a follow-up study. Lastly, although the SNPs that might cause pleiotropy were identified and removed with the outlier test in the MR-PRESSO analysis, there was still no evidence for the causal effect of IBD, UC, and CD on IgAN from the MR-Egger analysis. Hence, more advanced MR methods have to be developed for the causal estimate after suppressing the interference of pleiotropy.

In conclusion, we found a positive causal relationship of IBD, UC, and CD with IgAN at the genetic level by using two-sample MR analysis. However, there was not sufficient evidence for the causal effect of IgAN on IBD, UC, and CD. The findings of this study should remind clinicians that regular monitoring of renal function in patients with IBD should not be ignored and that optimized treatment for IBD could improve prognosis in patients with IgAN. However, additional studies are required to investigate and elucidate the potential mechanistic links between IBD and IgAN.

## Data Availability

The original contributions presented in the study are included in the article/Supplementary Material; further inquiries can be directed to the corresponding author.

## Abbreviations

CD, Crohn’s disease; CI, confidence intervals; ESKD, end-stage kidney disease; Gd-IgA1, galactose-deficient IgA1; GWAS, genome-wide association study; IBD, inflammatory bowel disease; IgAN, immunoglobulin A nephropathy; IV, instrumental variable; IVW, inverse-variance weighting; MR, Mendelian randomization; MR-Egger, Mendelian randomization–Egger; SNP, single-nucleotide polymorphism; UC, ulcerative colitis.
