# Supplementary material for: Causal association between inflammatory bowel disease and IgA nephropathy: A bidirectional two-sample Mendelian randomization study
Source: Front Genet. 2022 Nov 16;13:1002928. doi: 10.3389/fgene.2022.1002928 (PMC9710718; doi:10.3389/fgene.2022.1002928)
Supplement: Supplementary file 1 [file DataSheet1.PDF]

Supplemental Figures

Figure S1: Scatter plot of SNPs associated with IBD and IgA nephropathy.
Figure S2: Forest plot of SNPs associated with IBD and IgA nephropathy.
Figure S3: Funnel plot of SNPs associated with IBD and IgA nephropathy.
Figure S4: Leave-one-out analysis of SNPs associated with IBD and IgA nephropathy.
Figure S5: Scatter plot of SNPs associated with UC and IgA nephropathy.
Figure S6: Forest plot of SNPs associated with UC and IgA nephropathy.
Figure S7: Funnel plot of SNPs associated with UC and IgA nephropathy.
Figure S8: Leave-one-out analysis of SNPs associated with UC and IgA nephropathy.
Figure S9: Scatter plot of SNPs associated with CD and IgA nephropathy.
Figure S10: Forest plot of SNPs associated with CD and IgA nephropathy.
Figure S11: Funnel plot of SNPs associated with CD and IgA nephropathy.
Figure S12: Leave-one-out analysis of SNPs associated with CD and IgA nephropathy.

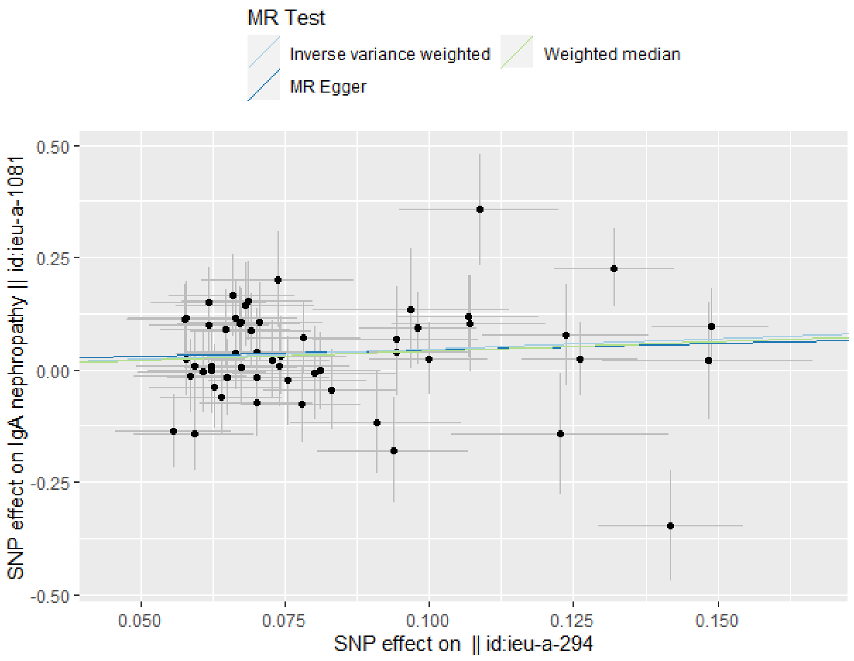

Figure S1: Scatter plot of SNPs associated with IBD and IgA nephropathy.

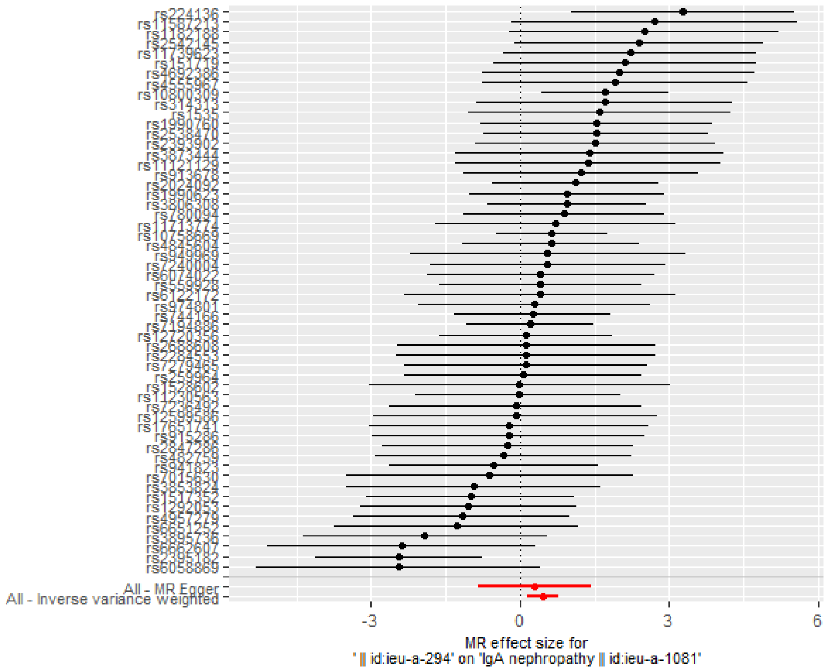

Figure S2: Forest plot of SNPs associated with IBD and IgA nephropathy.

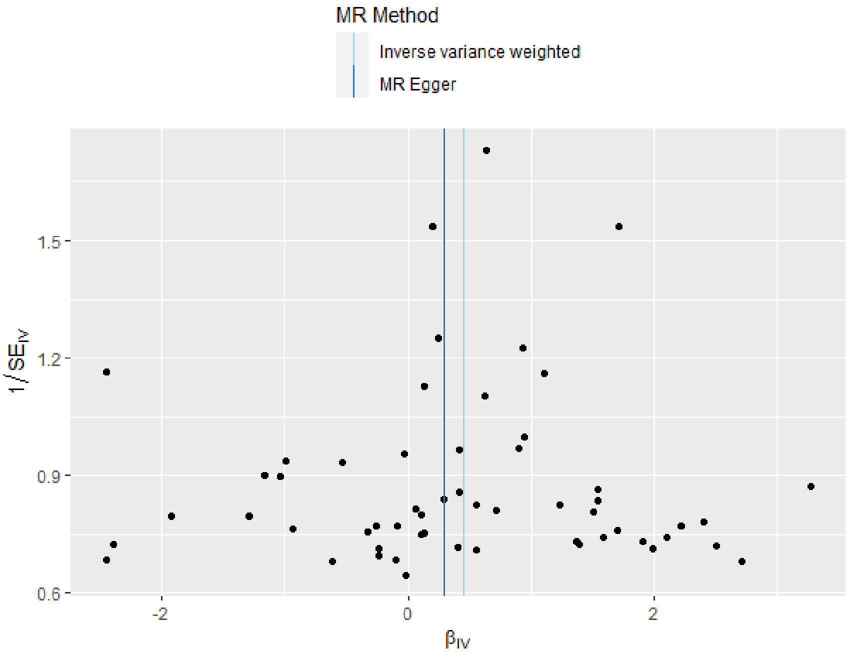

Figure S3: Funnel plot of SNPs associated with IBD and IgA nephropathy.

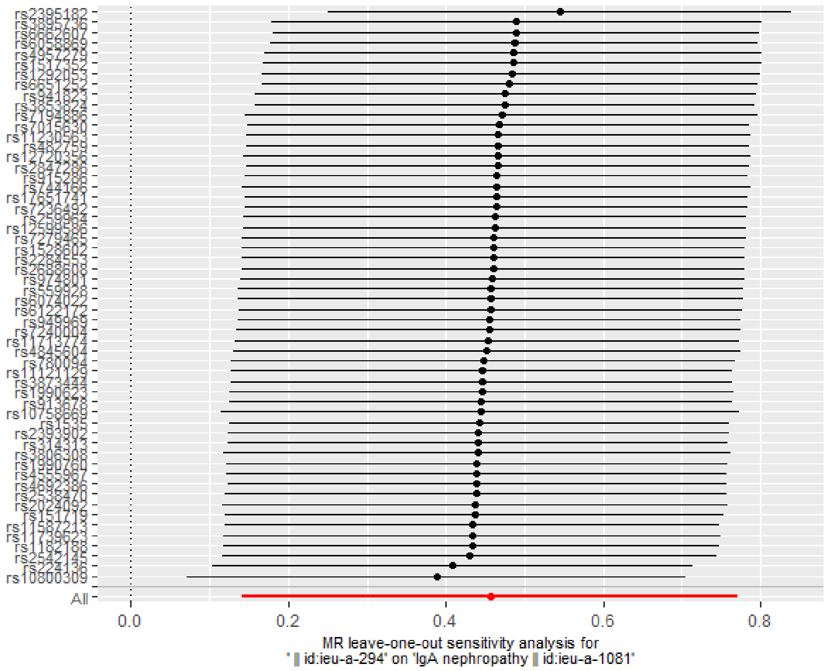

Figure S4: Leave-one-out analysis of SNPs associated with IBD and IgA nephropathy.

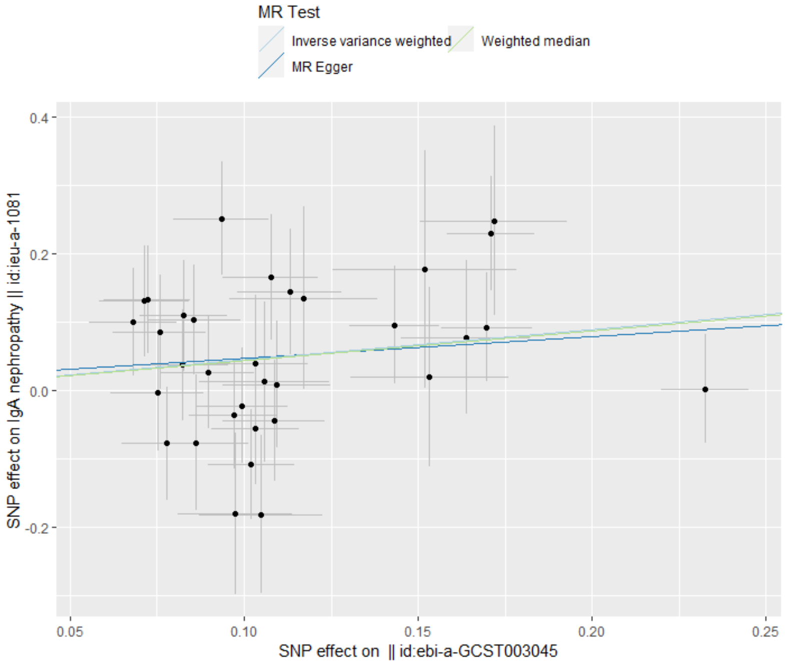

Figure S5: Scatter plot of SNPs associated with UC and IgA nephropathy.

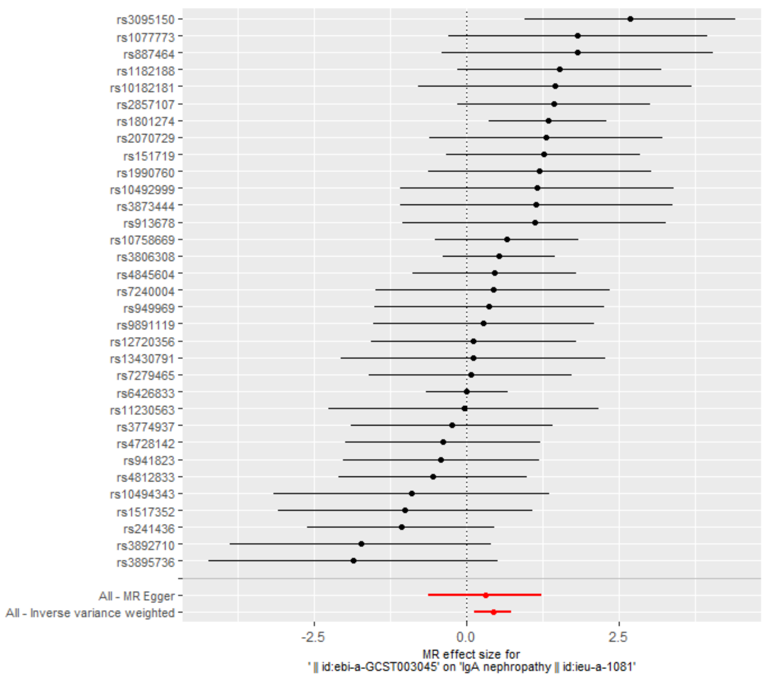

Figure S6: Forest plot of SNPs associated with UC and IgA nephropathy.

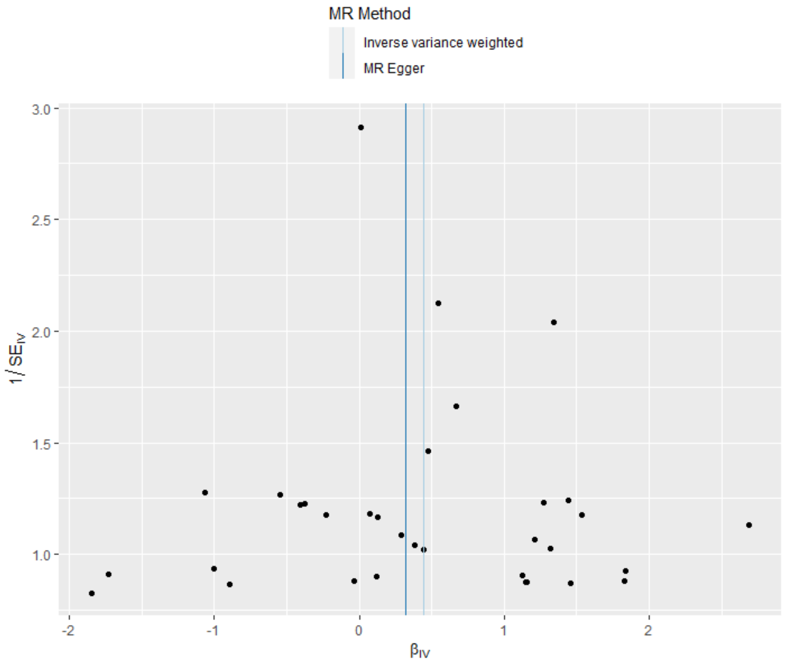

Figure S7: Funnel plot of SNPs associated with UC and IgA nephropathy.

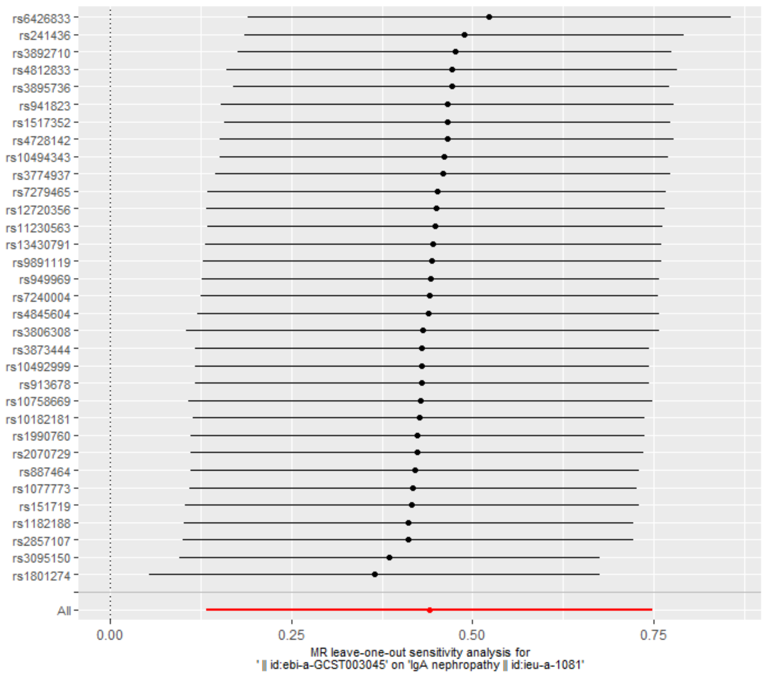

Figure S8: Leave-one-out analysis of SNPs associated with UC and IgA nephropathy.

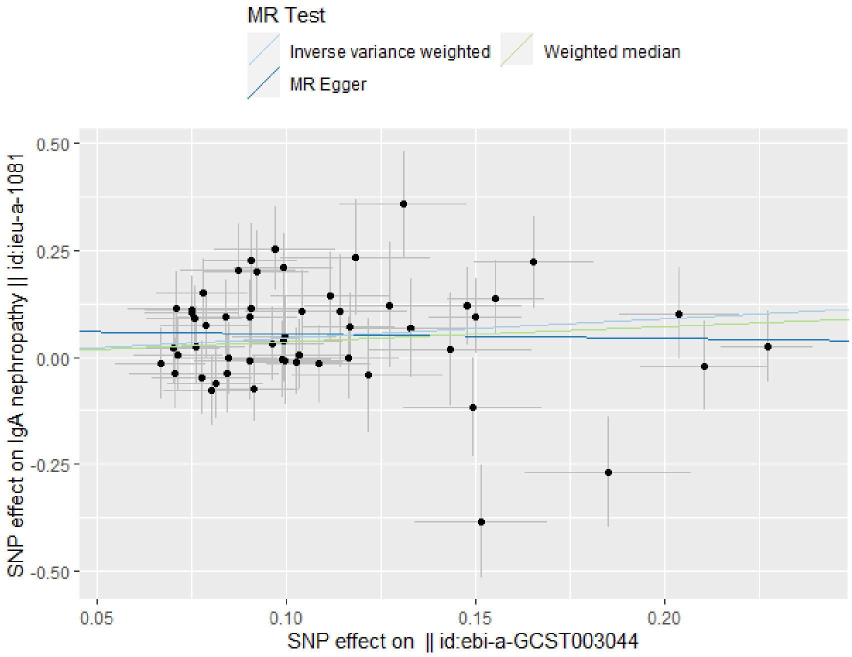

Figure S9: Scatter plot of SNPs associated with CD and IgA nephropathy.

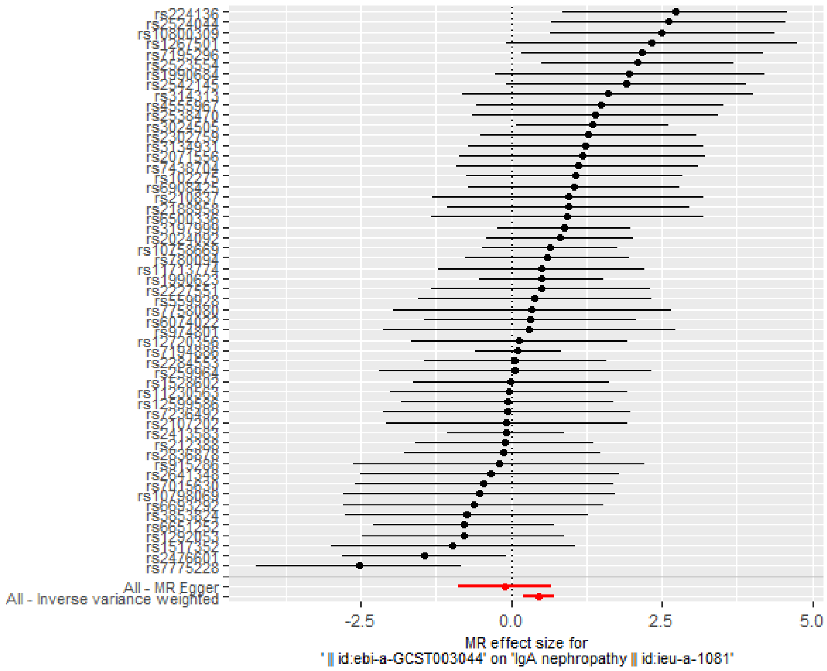

Figure S10: Forest plot of SNPs associated with CD and IgA nephropathy.

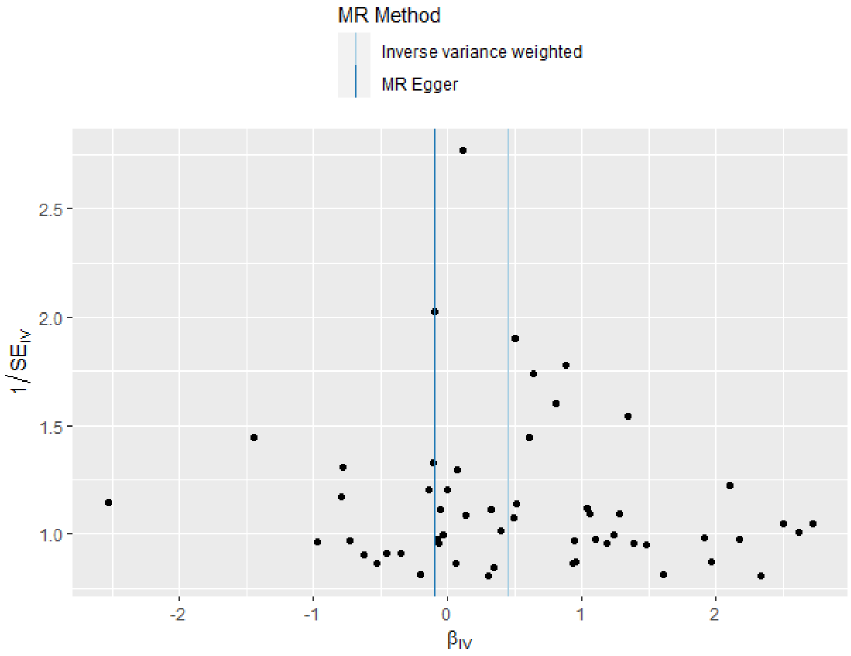

Figure S11: Funnel plot of SNPs associated with CD and IgA nephropathy.

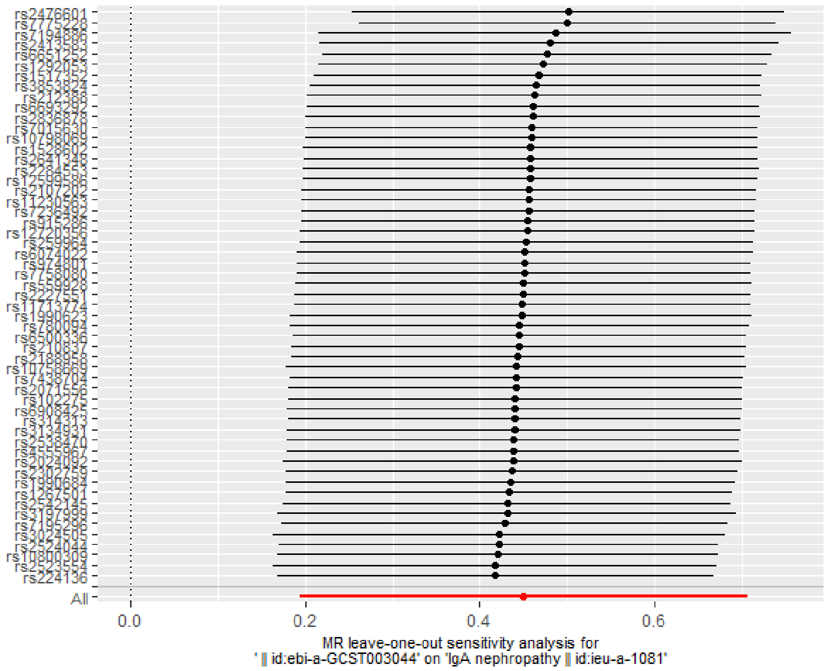

Figure S12: Leave-one-out analysis of SNPs associated with CD and IgA nephropathy.
